# Complete Genome Sequence of *Escherichia coli* ML35

**DOI:** 10.1128/genomeA.00034-18

**Published:** 2018-02-15

**Authors:** Angeline Casale, Stephanie Clark, Melissa Grasso, Marta Kryschuk, Lukas Ritzer, Madyson Trudeau, Laura E. Williams

**Affiliations:** a Department of Biology, Providence College, Providence, Rhode Island, USA

## Abstract

We report here the complete genome sequence of *Escherichia coli* strain ML35. We assembled PacBio reads into a single closed contig with 169× mean coverage and then polished this contig using Illumina MiSeq reads, yielding a 4,918,774-bp sequence with 50.8% GC content.

## GENOME ANNOUNCEMENT

*Escherichia coli* strain ML35 was isolated during studies of *lac* operon gene expression in the 1950s (1). ML35 does not synthesize lactose permease, but it constitutively expresses β-galactosidase (2). Since its isolation, ML35 has been used in a variety of experiments, including the investigation of interactions between *E. coli* and predatory bacteria (3). Williams and coworkers (4) are using ML35 and other *E. coli* strains to test the prey range of predatory bacteria. Comparative genomics will help us understand how genome variation within a prey species impacts variation in predation phenotypes.

We extracted genomic DNA from 3 ml of overnight culture grown in Trypticase soy broth at 37°C using the Wizard genomic DNA purification kit (Promega). Aliquots were used by the University of Maryland Institute for Genome Sciences to construct a PacBio library and by the University of Rhode Island Genomics and Sequencing Center to construct an Illumina library. Sequencing on a PacBio RS II instrument using P6-C4 chemistry yielded 93,133 subreads, with an *N*_50_ value of 12,583 bp, from two single-molecule real-time (SMRT) cells. For *de novo* assembly, we launched an Amazon EC2 instance of SMRT Portal version 2.3.0 and used the Hierarchical Genome Assembly Process version 3 (HGAP3) (5) with an estimated genome size 4.5 Mb and a target coverage of 30×. This generated contigs of 4,964,530 bp and 18,915 bp, with 169× and 18× mean coverages, respectively. The small contig is highly similar to regions of the large contig. Combined with its low coverage, this suggests that the small contig is an assembly artifact; therefore, we discarded it. To circularize the large contig, we used Gepard (6) to visualize overlap between the ends of the contig and BLAST (7) and EMBOSS extractseq (8) to specify coordinates and trim overlap, thereby generating a closed 4,918,091-bp contig.

To polish the closed contig, we processed 2 × 250-bp Illumina MiSeq reads using SolexaQA++ version 3.1.4 (9). We removed bases that had a quality score of <13 with DynamicTrim and then discarded reads that had <100 bp with LengthSort. This yielded 5,366,007 read pairs. Using the Burrows-Wheeler aligner “mem” (BWA-mem) algorithm version 0.7.13 (10), we mapped 94.8% of these reads to the closed contig. We sorted and indexed the alignment file with SAMtools (11) and then used Pilon version 1.22 (12) to identify and correct 717 small indels, yielding a corrected 4,918,780-bp contig. To confirm this sequence, we used the same Illumina MiSeq reads and DynamicTrim quality score cutoff but adjusted the LengthSort cutoff to 75 bp. After aligning these reads to the corrected contig, Pilon identified eight discrepancies, which we manually examined and corrected to generate the final genome sequence of 4,918,774 bp with 50.8% GC content.

Annotation with the Prokaryotic Genome Annotation Pipeline (PGAP) predicted 4,782 protein-coding sequences, 757 of which are annotated as hypothetical proteins, along with 95 tRNAs and 7 rRNA operons. By comparing the ML35 genome to that of *E. coli* MG1655 (GenBank accession no. NC_000913), we identified an 11-bp insertion in ML35’s *lacY* gene that causes a frameshift and a nonsynonymous substitution in ML35’s *lacI* gene that causes a V24E replacement, which is reported to impact the repressor protein function (13). These mutations may explain the Lac phenotype observed for ML35.

### Accession number(s).

This complete genome sequence has been deposited in GenBank under the accession no. CP025747. The version described in this paper is the first version, CP025747.1.
